# Operation and Prognosis of Lingual Cancer

**Published:** 1890-02

**Authors:** 


### Operation and Prognosis of Lingual Cancer.

During the past thirteen years (KRAUSE *Ctbl. f. Chirurgie*) ninety-one extirpations of the tongue for cancer have been done in Volkmann's clinic. In fifty-six partial excisions the mortality was 0 per cent., in thirty-five complete ones 5-7 per cent. These favorable results induced Volkmann to publish the technique and after-treatment he pursues.

Volkmann does not tie the lingual artery, nor do tracheotomy. When it is possible to bring the tongue with its tumor in front of the teeth, he excises through the mouth; in the severe cases Langenbeck's method of temporary section of the lower maxilla is practiced by means of an ordinary saw. The palatol-glossal arch is severed each time, and later a drainage-tube is put into the tonsil-niche. After thorough extirpation, the wound is united by bringing the mucous membranes as near as possible together. The maxilla is united by silver wire, and produces either bony or ligamentous union; sometimes partial necrosis. The œsophageal tube is not used. Operation close to the epiglottis is declined. After the thirty-two severe cases, the average duration of life was twelve months; one man lived over six years. Among the fifty-six partial excisions seven remain alive; the shortest period of survival, eight months; the longest, six years.—*Times and Register.*

